# Supplementary material for: Adapting the Opening Minds Stigma Scale for Healthcare Providers to Measure Opioid-Related Stigma
Source: Pharmacy (Basel). 2024 Jul 9;12(4):105. doi: 10.3390/pharmacy12040105 (PMC11270195; doi:10.3390/pharmacy12040105)
Supplement: Supplementary file 1 [file pharmacy-12-00105-s001.zip › pharmacy-3058023-supplementary.pdf]

Version 1:

- I am more comfortable helping a person who has a physical illness than I am helping a person who has an opioid dependency.

1 = Strongly disagree, 2 = Disagree, 3 = Neither agree nor disagree, 4 = Agree and 5 = Strongly agree

- Despite my professional beliefs, I have negative reactions towards people who have an opioid dependency.

1 = Strongly disagree, 2 = Disagree, 3 = Neither agree nor disagree, 4 = Agree and 5 = Strongly agree

- There is little I can do to help people with an opioid dependency.

1 = Strongly disagree, 2 = Disagree, 3 = Neither agree nor disagree, 4 = Agree and 5 = Strongly agree

- More than half of people with an opioid dependency don't try hard enough to get better.

1 = Strongly disagree, 2 = Disagree, 3 = Neither agree nor disagree, 4 = Agree and 5 = Strongly agree

- Health care providers do not need to be advocates for people with an opioid dependency.

1 = Strongly disagree, 2 = Disagree, 3 = Neither agree nor disagree, 4 = Agree and 5 = Strongly agree

- I struggle to feel compassion for a person with an opioid dependency.

1 = Strongly disagree, 2 = Disagree, 3 = Neither agree nor disagree, 4 = Agree and 5 = Strongly agree

- If I were under treatment for an opioid dependency I would not disclose this to any of my colleagues.

1 = Strongly disagree, 2 = Disagree, 3 = Neither agree nor disagree, 4 = Agree and 5 = Strongly agree

- I would see myself as weak if I had an opioid dependency and could not fix it myself.

1 = Strongly disagree, 2 = Disagree, 3 = Neither agree nor disagree, 4 = Agree and 5 = Strongly agree

- I would be reluctant to seek help if I had an opioid dependency.

1 = Strongly disagree, 2 = Disagree, 3 = Neither agree nor disagree, 4 = Agree and 5 = Strongly agree

- If I had an opioid dependency, I would tell my friends.

5 = Strongly disagree, 4 = Disagree, 3 = Neither agree nor disagree, 2 = Agree and 1 = Strongly agree

- If a colleague with whom I work told me they have a managed opioid dependency, I would be as willing to work with them.

5 = Strongly disagree, 4 = Disagree, 3 = Neither agree nor disagree, 2 = Agree and 1 = Strongly agree

- Employers should hire a person with a managed opioid dependence if they are the best person for the job.

5 = Strongly disagree, 4 = Disagree, 3 = Neither agree nor disagree, 2 = Agree and 1 = Strongly agree

- I would still go to a physician if I knew that the physician had been treated for an opioid dependency.

5 = Strongly disagree, 4 = Disagree, 3 = Neither agree nor disagree, 2 = Agree and 1 = Strongly agree

- I would not want a person with an opioid dependency, even if it were appropriately managed, to work with children.

1 = Strongly disagree, 2 = Disagree, 3 = Neither agree nor disagree, 4 = Agree and 5 = Strongly agree

- I would not mind if a person with an opioid dependency lived next door to me.

5 = Strongly disagree, 4 = Disagree, 3 = Neither agree nor disagree, 2 = Agree and 1 = Strongly agree

Version 2:

- I am more comfortable helping a person who has a physical illness than I am helping a person who has an opioid use disorder.

1 = Strongly disagree, 2 = Disagree, 3 = Neither agree nor disagree, 4 = Agree and 5 = Strongly agree

- Despite my professional beliefs, I have negative reactions towards people who have an opioid use disorder.

1 = Strongly disagree, 2 = Disagree, 3 = Neither agree nor disagree, 4 = Agree and 5 = Strongly agree

- There is little I can do to help people with an opioid use disorder.

1 = Strongly disagree, 2 = Disagree, 3 = Neither agree nor disagree, 4 = Agree and 5 = Strongly agree

- More than half of people with an opioid use disorder don't try hard enough to get better.

1 = Strongly disagree, 2 = Disagree, 3 = Neither agree nor disagree, 4 = Agree and 5 = Strongly agree

- Health care providers do not need to be advocates for people with an opioid use disorder.

1 = Strongly disagree, 2 = Disagree, 3 = Neither agree nor disagree, 4 = Agree and 5 = Strongly agree

- I struggle to feel compassion for a person with an opioid use disorder.

1 = Strongly disagree, 2 = Disagree, 3 = Neither agree nor disagree, 4 = Agree and 5 = Strongly agree

- If I were under treatment for an opioid use disorder I would not disclose this to any of my colleagues.

1 = Strongly disagree, 2 = Disagree, 3 = Neither agree nor disagree, 4 = Agree and 5 = Strongly agree

- I would see myself as weak if I had an opioid use disorder and could not fix it myself.

1 = Strongly disagree, 2 = Disagree, 3 = Neither agree nor disagree, 4 = Agree and 5 = Strongly agree

- I would be reluctant to seek help if I had an opioid use disorder.

1 = Strongly disagree, 2 = Disagree, 3 = Neither agree nor disagree, 4 = Agree and 5 = Strongly agree

- If I had an opioid use disorder, I would tell my friends.

5 = Strongly disagree, 4 = Disagree, 3 = Neither agree nor disagree, 2 = Agree and 1 = Strongly agree

- If a colleague with whom I work told me they have a managed opioid use disorder, I would be as willing to work with them.

5 = Strongly disagree, 4 = Disagree, 3 = Neither agree nor disagree, 2 = Agree and 1 = Strongly agree

- Employers should hire a person with a managed opioid use disorder if they are the best person for the job.

5 = Strongly disagree, 4 = Disagree, 3 = Neither agree nor disagree, 2 = Agree and 1 = Strongly agree

- I would still go to a physician if I knew that the physician had been treated for an opioid use disorder.

5 = Strongly disagree, 4 = Disagree, 3 = Neither agree nor disagree, 2 = Agree and 1 = Strongly agree

- I would not want a person with an opioid use disorder, even if it were appropriately managed, to work with children.

1 = Strongly disagree, 2 = Disagree, 3 = Neither agree nor disagree, 4 = Agree and 5 = Strongly agree

- I would not mind if a person with an opioid use disorder lived next door to me.

5 = Strongly disagree, 4 = Disagree, 3 = Neither agree nor disagree, 2 = Agree and 1 = Strongly agree

Version 3:

- I am more comfortable helping a person who has a physical illness than I am helping a person who has an opioid addiction.

1 = Strongly disagree, 2 = Disagree, 3 = Neither agree nor disagree, 4 = Agree and 5 = Strongly agree

- Despite my professional beliefs, I have negative reactions towards people who have an opioid addiction.

1 = Strongly disagree, 2 = Disagree, 3 = Neither agree nor disagree, 4 = Agree and 5 = Strongly agree

- There is little I can do to help people with an opioid addiction.

1 = Strongly disagree, 2 = Disagree, 3 = Neither agree nor disagree, 4 = Agree and 5 = Strongly agree

- More than half of people with an opioid addiction don’t try hard enough to get better.

1 = Strongly disagree, 2 = Disagree, 3 = Neither agree nor disagree, 4 = Agree and 5 = Strongly agree

- Health care providers do not need to be advocates for people with an opioid addiction.

1 = Strongly disagree, 2 = Disagree, 3 = Neither agree nor disagree, 4 = Agree and 5 = Strongly agree

- I struggle to feel compassion for a person with an opioid addiction.

1 = Strongly disagree, 2 = Disagree, 3 = Neither agree nor disagree, 4 = Agree and 5 = Strongly agree

- If I were under treatment for an opioid addiction I would not disclose this to any of my colleagues.

1 = Strongly disagree, 2 = Disagree, 3 = Neither agree nor disagree, 4 = Agree and 5 = Strongly agree

- I would see myself as weak if I had an opioid addiction and could not fix it myself.

1 = Strongly disagree, 2 = Disagree, 3 = Neither agree nor disagree, 4 = Agree and 5 = Strongly agree

- I would be reluctant to seek help if I had an opioid addiction.

1 = Strongly disagree, 2 = Disagree, 3 = Neither agree nor disagree, 4 = Agree and 5 = Strongly agree

- If I had an opioid addiction, I would tell my friends.

5 = Strongly disagree, 4 = Disagree, 3 = Neither agree nor disagree, 2 = Agree and 1 = Strongly agree

- If a colleague with whom I work told me they have a managed opioid addiction, I would be as willing to work with them.

5 = Strongly disagree, 4 = Disagree, 3 = Neither agree nor disagree, 2 = Agree and 1 = Strongly agree

- Employers should hire a person with a managed opioid addiction if they are the best person for the job.

5 = Strongly disagree, 4 = Disagree, 3 = Neither agree nor disagree, 2 = Agree and 1 = Strongly agree

- I would still go to a physician if I knew that the physician had been treated for an opioid addiction.

5 = Strongly disagree, 4 = Disagree, 3 = Neither agree nor disagree, 2 = Agree and 1 = Strongly agree

- I would not want a person with an opioid addiction, even if it were appropriately managed, to work with children.

1 = Strongly disagree, 2 = Disagree, 3 = Neither agree nor disagree, 4 = Agree and 5 = Strongly agree

- I would not mind if a person with an opioid addiction lived next door to me.

5 = Strongly disagree, 4 = Disagree, 3 = Neither agree nor disagree, 2 = Agree and 1 = Strongly agree

Version 4:

- I am more comfortable helping a person who has a physical illness than I am helping a person who has an opioid misuse/use disorder.

1 = Strongly disagree, 2 = Disagree, 3 = Neither agree nor disagree, 4 = Agree and 5 = Strongly agree

- Despite my professional beliefs, I have negative reactions towards people who have an opioid misuse/use disorder.

1 = Strongly disagree, 2 = Disagree, 3 = Neither agree nor disagree, 4 = Agree and 5 = Strongly agree

- There is little I can do to help people with an opioid misuse/use disorder.

1 = Strongly disagree, 2 = Disagree, 3 = Neither agree nor disagree, 4 = Agree and 5 = Strongly agree

- More than half of people with an opioid misuse/use disorder don’t try hard enough to get better.

1 = Strongly disagree, 2 = Disagree, 3 = Neither agree nor disagree, 4 = Agree and 5 = Strongly agree

- Health care providers do not need to be advocates for people with an opioid misuse/use disorder.

1 = Strongly disagree, 2 = Disagree, 3 = Neither agree nor disagree, 4 = Agree and 5 = Strongly agree

- I struggle to feel compassion for a person with an opioid misuse/use disorder.

1 = Strongly disagree, 2 = Disagree, 3 = Neither agree nor disagree, 4 = Agree and 5 = Strongly agree

- If I were under treatment for an opioid misuse/use disorder I would not disclose this to any of my colleagues.

1 = Strongly disagree, 2 = Disagree, 3 = Neither agree nor disagree, 4 = Agree and 5 = Strongly agree

- I would see myself as weak if I had an opioid misuse/use disorder and could not fix it myself.

1 = Strongly disagree, 2 = Disagree, 3 = Neither agree nor disagree, 4 = Agree and 5 = Strongly agree

- I would be reluctant to seek help if I had an opioid misuse/use disorder.

1 = Strongly disagree, 2 = Disagree, 3 = Neither agree nor disagree, 4 = Agree and 5 = Strongly agree

- If I had an opioid misuse/use disorder, I would tell my friends.

5 = Strongly disagree, 4 = Disagree, 3 = Neither agree nor disagree, 2 = Agree and 1 = Strongly agree

- If a colleague with whom I work told me they have a managed opioid misuse/use disorder, I would be as willing to work with them.

5 = Strongly disagree, 4 = Disagree, 3 = Neither agree nor disagree, 2 = Agree and 1 = Strongly agree

- Employers should hire a person with a managed opioid misuse/use disorder if they are the best person for the job.

5 = Strongly disagree, 4 = Disagree, 3 = Neither agree nor disagree, 2 = Agree and 1 = Strongly agree

- I would still go to a physician if I knew that the physician had been treated for an opioid misuse/use disorder.

5 = Strongly disagree, 4 = Disagree, 3 = Neither agree nor disagree, 2 = Agree and 1 = Strongly agree

- I would not want a person with an opioid misuse/use disorder, even if it were appropriately managed, to work with children.

1 = Strongly disagree, 2 = Disagree, 3 = Neither agree nor disagree, 4 = Agree and 5 = Strongly agree

- I would not mind if a person with an opioid misuse/use disorder lived next door to me.

5 = Strongly disagree, 4 = Disagree, 3 = Neither agree nor disagree, 2 = Agree and 1 = Strongly agree

Question included in all versions:

Please rank the four terms in order from least (1) to most (4) stigmatizing

- Opioid dependency
- Opioid use disorder
- Opioid addiction
- Opioid misuse/use disorder

Please expand on the rationale for your ranking of the four terms above:

- Open text

Please add your UW email for the participation grade below:

- Open text
